# Inverse Association of Plasma IgG Antibody to *Aggregatibacter actinomycetemcomitans* and High C-Reactive Protein Levels in Patients with Metabolic Syndrome and Periodontitis

**DOI:** 10.1371/journal.pone.0148638

**Published:** 2016-02-12

**Authors:** Supanee Thanakun, Suchaya Pornprasertsuk-Damrongsri, Misa Gokyu, Hiroaki Kobayashi, Yuichi Izumi

**Affiliations:** 1 Department of Oral Medicine and Periodontology, Faculty of Dentistry, Mahidol University, Bangkok, Thailand; 2 Department of Oral and Maxillofacial Radiology, Faculty of Dentistry, Mahidol University, Bangkok, Thailand; 3 Department of Periodontology, Graduate School of Medical and Dental Sciences, Tokyo Medical and Dental University, Tokyo, Japan; 4 Global Center of Excellence Program for Tooth and Bone Research, Tokyo Medical and Dental University, Tokyo, Japan; Medical University of South Carolina, UNITED STATES

## Abstract

The association between clinically diagnosed periodontitis, a common chronic oral infection, and metabolic syndrome has been previously reported. The aim of this study was to investigate the association of plasma IgG levels against *Aggregatibacter actinomycetemcomitans*, *Porphyromonas gingivalis*, *and Prevotella intermedia*, C-reactive protein, and periodontal status with metabolic syndrome. Plasma IgG levels and C-reactive protein were measured by enzyme-linked immunosorbent assay, and salivary levels of *A*. *actinomycetemcomitans* and *P*. *gingivalis* were determined by quantitative real-time polymerase chain reaction. Among 127 individuals aged 35–76 years, 57 participants had metabolic syndrome and severe periodontitis, 25 had metabolic syndrome and an absence of severe periodontitis, 17 healthy individuals had severe periodontitis, and 28 healthy individuals were without severe periodontitis. Patients with metabolic syndrome had reduced humoral immune response to *A*. *actinomycetemcomitans* (*p* = 0.008), regardless of their salivary levels or periodontitis status compared with healthy participants. The IgG antibody response to *P*. *gingivalis*, regardless of their salivary levels or participants’ health condition, was significantly higher in severe periodontitis patients (*p*<0.001). Plasma IgG titers for *P*. *intermedia* were inconsistent among metabolic syndrome or periodontal participants. Our results indicate that the presence of lower levels of IgG antibodies to *A*. *actinomycetemcomitans* (OR = 0.1; 95%CI 0.0–0.7), but not *P*. *gingivalis*, a severe periodontitis status (OR = 7.8; 95%CI 1.1–57.0), high C-reactive protein levels (OR = 9.4; 95%CI 1.0–88.2) and body mass index (OR = 3.0; 95%CI 1.7–5.2), are associated with the presence of metabolic syndrome. The role of the decreased IgG antibody response to *A*. *actinomycetemcomitans*, increased C-reactive protein levels on the association between periodontal disease and metabolic syndrome in a group of Thai patients is suggested.

## Introduction

Evidence for an association between periodontitis; one of the most common oral infections, and metabolic syndrome (MS); the predisposing condition to coronary heart disease (CHD), has been recently reported in the literature [1]. Our previous study also showed that patients with untreated MS had more severe periodontal disease than healthy individuals [2]. The biological mechanism for the association of MS with periodontitis is not well known. However, many inflammatory cytokines are postulated to play an important role [3].

Periodontopathic bacteria *Aggregatibacter actinomycetemcomitans* (*A*. *actinomycetemcomitans*), *Porphyromonas gingivalis* (*P*. *gingivalis*), *and Prevotella intermedia* (*P*. *intermedia*) can trigger host immunological inflammatory responses, both locally and systemically, and generate secretion of many inflammatory cytokines. Cytokines cause local inflammation leading to periodontal destruction, and these cytokines affect the entire body, contributing to low-grade systemic and vascular inflammation, and promoting atherosclerosis [4]. The systemic immunological response to periodontitis can be measured as increased plasma antibody levels against these periodontopathic bacteria. Ueno et al. reported that high plasma antibody levels to *A*. *actinomycetemcomitans*, *P*. *gingivalis* and *P*. *intermedia* were associated with an increased risk of CHD [5]. High levels of Immunoglobulin G (IgG) antibody titers to *P*. *gingivalis* are also observed in periodontitis patients [6]. With this association with the chronic inflammatory response, it is postulated that periodontitis may enhance atherosclerosis in patients with MS, leading to early CHD.

High levels of C-reactive protein (CRP), a sensitive and dynamic systemic marker of inflammation, have been reported in patients with MS [7,8]. Several studies have also reported elevated CRP levels in periodontitis patients [9–11]. Therefore, the role of CRP needs to be further evaluated by considering the effects of other factors, such as oral infections that could modify CRP levels and maintain a low-grade systemic inflammation.

The antibody response to periodontopathic bacteria and levels of CRP could be associated with MS and periodontitis. We hypothesized that pro-inflammatory cytokines produced in MS may modulate the inflammatory response to periodontopathic bacteria and enhance severity of periodontal disease in clinical setting. However, no reports have investigated the association between CRP levels, IgG antibody titers to *A*. *actinomycetemcomitans*, *P*. *gingivalis*, and *P*. *intermedia*, salivary bacteria levels, and the clinical periodontal condition of severe periodontitis in a sample population of MS patients compared with healthy individuals. Patients with MS are an important group to study because they may benefit from treatment interventions for periodontal disease and prevent later progression of CHD. The purpose of this study is therefore to evaluate the role of the IgG antibody response to periodontopathic bacteria in the association of MS with periodontitis in a group of Thai patients. We determined the levels of IgG to three major periodontal pathogens, *A*. *actinomycetemcomitans*, *P*. *gingivalis*, and *P*. *intermedia*, and CRP in patients with MS with and without periodontitis. We examined whether CRP or plasma IgG antibody levels to these periodontal pathogens were associated with MS. The study also evaluated whether titers of IgG antibody to periodontopathic bacteria were correlated with their amount in saliva and CRP levels in patients with MS and periodontitis.

## Materials and Methods

### Study participants and assessment of MS

Patients who attended the Golden Jubilee Medical Center, Mahidol University for medical health checkup during July 2012-August 2013 were assessed for MS. The Ethics Committee of Mahidol University and Tokyo Medical and Dental University approved this study, and the protocol conformed to the Declaration of Helsinki (reference number: MU-DT/TY-IRB 2012/042.0911, TMDU-IRB 2012/1108.860). Participants were between 35–76 years of age. Waist circumference (WC), levels of triglyceride (TG), high density lipoprotein cholesterol (HDL-C), fasting plasma glucose (FPG), blood pressure (BP), and body mass index (BMI) were evaluated from medical records. MS was diagnosed when three of the following five factors were present. These included (1) elevated WC (≥85 cm in Thai males and ≥80 cm in Thai females), (2) elevated TG levels (≥150 mg/dL), (3) reduced HDL-C levels (<40 mg/dL in males and <50 mg/dL in females), (4) elevated BP (systolic BP≥ 130 or diastolic BP≥85 mmHg), and (5) elevated FPG levels (≥ 100 mg/dL). Although drug treatment for abnormalities in items (2) to (5) is an alternate indicator [12], patients treated for MS with these drugs were excluded from this study. Exclusion criteria included (1) known other systemic diseases, (2) history and/or presence of other infections and (3) systemic antibiotics, immunosuppressive drugs or periodontal treatment in the 6 months prior to sample collection. All participants were fully informed before completing their written consent document. Demographic data were collected. One hundred thirty nine individuals with untreated MS who met inclusion criteria and desired to participate were enrolled for the current study. Oral examination, blood and saliva collection were subsequently taken.

### Assessment of periodontitis

Full-mouth periodontal examinations were performed by one examiner (ST) with a standardized method as described previously [2]. Bleeding on probing (BOP), probing depth (PD), and clinical attachment level (CAL) were recorded at six sites around each tooth to accurately diagnose periodontal disease. Panoramic extra-oral radiographs (Planmeca Proline XC, Helsinki, Finland) were obtained for all participants. Alveolar bone resorption was measured on the proximal surface of each tooth radiographically by an oral and maxillofacial radiologist (SD) and mean levels were calculated from six teeth according to Beckstrom et al. [13]. Periodontitis was diagnosed when BOP, PD ≥3 mm and CAL≥1 mm were presented in at least 1 site. Severity is categorized on the basis of the amount of maximum CAL. Severe periodontitis was diagnosed according to the clinical criteria suggested by Armitage where maximum CAL>4 mm is classified as severe periodontitis [14].

### Assessment of confounders

To obtain data regarding potential confounders, information on occupation (government officer/employee, commerce, miscellaneous), education level (lower or university Bachelor/higher), exercise (none, sometimes [≤2 times per week], yes [≥3 times a week]) and personal habits (smoking and alcohol consumption: never, former, and current), as well as oral health behavior (frequency of tooth brushing: ≤1 time or ≥2 times daily) was collected by interview using structured questionnaires.

### Assays for plasma IgG antibody to three periodontopathic bacteria and CRP levels

On the same date that peripheral blood was collected for a health examination, a portion was centrifuged, and 0.5-ml aliquots of plasma were obtained and frozen (−80°C) for subsequent analyses.

Plasma IgG antibody and CRP measurement was done by enzyme-linked immunosorbent assay (ELISA). IgG antibody for the following three periodontopathic bacteria: *A*. *actinomycetemcomitans* ATCC 43718, *P*. *gingivalis* ATCC 33277 and *P*. *intermedia* ATCC 25611 were analyzed using ELISA kit (Toagosei Co., Tokyo, Japan). According to the manufacturer’s manual, these three periodontopathic bacteria were obtained from the American Type culture Collection (Rockville, MD, USA). *A*. *actinomycetemcomitans* was inoculated on a trypticase soy bacitracin vancomycin (TSBV) agar plate (containing 1 g/L yeast extract, 100 ml/L horse serum, 75 mg/L bacitracin and 5 mg/L vancomycin). The TSBV plate, which is a selective medium for *A*. *actinomycetemcomitans*, was incubated at 37°C for one week in a 10%- CO2, 90%—air environment. *P*. *gingivalis* and *P*. *intermedia* were inoculated on trypticase soy agar plates (supplemented with 50 ml/L horse serum, 5 mg/L hemin and 0.1 mg/L vitamin K1) and were incubated at 37°C for one week in an anaerobic condition (10%—H2, 10%—C02, 80%—N2). *A*. *actinomycetemcomitans* on TSBV plates was identified based on colonial morphology such as adherence to the agar surface and star-like inner structure together with catalase positive reaction. Greenish-black-pigmented and benzoyl-DL-arginine-naphthylamide (BANA) positive colonies were identified as *P*. *gingivitis*. The BANA test was performed using BANAPERIO (Hakusui Trading Co., Osaka, Japan). *P*. *intermedia* was also identified based on colonial morphology (circular, convex and shiny gray/brown to black colonies). Some isolates of these bacteria were also confirmed by the polymerase chain reaction. A single bacterial colony was transferred into a brain heart infusion broth (for *A*. *actinomycetemcomitans*) or a trypticase soy broth (supplemented with 5 mg/L hemin and 0.1 mg/L vitamin K1: for *P*. *gingivalis* and *P*. *intermedia*). The bacteria were cultured anaerobically in one liters of broth for 48 to 72h and used for preparation of bacterial antigens. Subsequently, antigens were prepared by cold ultrasonication of washed bacterial cells. The sonicated supernatants were dialyzed against distilled water, lyophilized, and stored at—20°C until used [15]. The 96-well microtiter plates (EIA plate, Costar, Cambridge, MA, USA) were later coated with sonicated whole-cell extracts.

Plasma samples were thawed at room temperature and were diluted 400-fold. Six different concentrations of reference solution were prepared. Subsequently, 100 μL of the diluted plasma sample and reference solution were added to each well in duplicate and the plates were incubated for 1 hour at room temperature. Following incubation, the plates were washed four times with 300 μL/well of 0.05% Tween-20/PBS. One hundred microliters of alkaline phosphatase-conjugated goat anti-human IgG (Sigma Chemical Co., NY, USA) was added and incubated at room temperature for an hour. Washing with 300 μL/well of 0.05% Tween-20/PBS was repeated four times. After adding 100 μL of enzyme substrate solution (tetramethylbenzidine) (DAKO, Sigma) and a 30-minute incubation at 24°C in the dark, the reaction was stopped with 100 μL of 2 N sulfuric acid. The absorbance of each well was read immediately thereafter using a Microplate Reader (SOFTMax^TM^ Molecular Devices Corp., CA, USA) at 450 nm with a 650-nm reference wavelength. Individual plasma antibody levels of periodontal pathogens (U/mL) were calculated from the reference curves of antibody concentrations for periodontal pathogens and the absorbance density.

The human CRP commercial ELISA kit (R&D^®^, Minneapolis, MN, USA) was used for CRP measurement. The assay was conducted according to the manufacturer’s instructions. The lower limit of the assay was 0.005 ng/mL. The recovery percentage of CRP ranged from 92–110%. Intra- and inter-assay coefficients of variation (CVs) were less than 10%. There was no cross-reactivity with other cytokines.

### Assays for levels of salivary *A*. *actinomycetemcomitans* and *P*. *gingivalis*

Unstimulated whole saliva samples were collected during the same visit for MS assessment and periodontal examination. Samples were kept in a freezer at –80°C until used for the extraction of bacterial DNA (QIAmp DNA Mini Kit; Valencia, CA, USA). We determined levels of *A*. *actinomycetemcomitans* and *P*. *gingivalis* by quantitative real-time polymerase chain reaction (qRT-PCR). Periodontopathogens were identified using qRT-PCR based on 16S rRNA genes. The PCR reaction mixture contained 12.5 μl of Premix ExTaq (Probe qPCR) (Takara-bio Inc., Shiga, Japan), 0.5 μl of 10 nM forward and reverse primers (*A*. *actinomycetemcomitans* and *P*. *gingivalis*), 0.5 μl of final concentration 10 nM TaqMan^®^Probe (*A*. *actinomycetemcomitans* and *P*. *gingivalis*), 7.2 μl of sterilized DNase- and RNase- free water, and 1.0 μl of sample template DNA. qRT-PCR was performed with a Thermal Cycler Dice Real Time System II (Takara-bio Inc.) with the following thermal profile and sets of primers (Takara-Bio) [16,17]:

One cycle at 95°C for 30 s followed by 40 cycles at 95°C each for 5 s, and at 60°C for 30 s for two-step PCR.

*A*. *actinomycetemcomitans* Forward: 5’-GTCATCATGGCCCTTACGAGTAG-3’

*A*. *actinomycetemcomitans* Reverse: 5’-CCCCATCGCTGGTTGGT-3’

*A*. *actinomycetemcomitans* Probe: FAM-ACACGTGCTACAATGGCGTATACAGAGGGT-TAMRA and

*P*. *gingivalis* Forward: 5’-TAGCTTGCTAAGGTCGATGG-3’,

*P*. *gingivalis* Reverse: 5’-CAAGTGTATGCGGTTTTAGT-3’

*P*. *gingivalis* Probe: FAM-TGCGTAACGCGTATGCAACTTGCC-TAMRA

The threshold cycle was based on the crossing point method. Artificial synthetic gene was used for making standard curve. All data were analyzed using Thermal Cycler Dice Real Time System Software (Takara-bio Inc.).

### Statistical analyses

All statistical analyses were performed with SPSS 16.0 software for Windows (SPSS Inc., Illinois, USA). A *p* value of <0.05 was considered statistically significant. The results were reported by MS-specific and periodontitis status distributions. The data were analyzed by chi-square, analysis of variance with Bonferroni post-hoc test or Kruskal–Wallis test where appropriate after normal distribution proven by Kolmogorov–Smirnov test. The Spearman’s correlation test was used to determine correlation between CRP and IgG antibody levels of each periodontopathic bacterium. Stepwise multiple regression analyses were performed to determine whether there were correlations among CRP, periodontopathic bacteria IgG antibody levels and the following independent variables: (1) MS: presence, 4–5 components or each MS components and (2) periodontal status: presence of severe periodontitis, percentage of bleeding sites, mean PD, CAL, and alveolar bone loss.

Crude odd ratios (ORs) and 95% confidence intervals (CI) for MS were estimated by the tertile levels of plasma IgG antibody to the three periodontopathic bacteria and CRP levels using a logistic regression model. Tertile cutoff points of each bacterium IgG antibody and CRP were based on the frequency distribution of all subjects: *A*. *actinomycetemcomitans* (<12,877.8, 12,877.8–33,989.8, >33,989.8 U/mL), *P*. *gingivalis* (<15,759.0, 15,759.0–57,193.7, >57,193.7 U/mL), *P*. *intermedia* (<129,297.3, 129,297.3–230,001.8, 230,001.8 U/mL) and CRP (<1,066.2, 1,066.2–2,306.4, >2,306.4 ng/mL). Adjusted ORs were computed by entering the potential confounder variables age, sex, and BMI into the logistic regression model.

## Results

### Characteristics of the study population

One hundred twenty seven individuals were finally included in the present study. Eighty-two patients (64.6%) were diagnosed with MS and had never received treatment. Forty-five healthy participants (35.4%) were recruited as a control group. The characteristics of the individuals are presented in Table 1. Patients with MS and severe periodontitis had a higher age than patients without severe periodontitis (*p* = 0.001). They also had higher PD and CAL than patients with MS but without severe periodontitis or healthy participants (*p*<0.001). Additionally, patients in this group had higher average percentage of bleeding and alveolar bone loss than patients without severe periodontitis (*p*<0.001 and *p* = 0.027, respectively; data not shown).

**Table 1:** Characteristics of individuals [median (first, third quartile), mean±S.D. or n (%)] according to the presence or absence of MS and severe periodontitis.

	Healthy participants		Patients with MS		p*
	(n = 45)		(n = 82)		
	Absence of	Presence of	Absence of	Presence of	
	severe periodontitis	severe periodontitis	severe periodontitis	severe periodontitis	
	(n = 28)	(n = 17)	(n = 25)	(n = 57)	
**Age (years)**	44.0 (38.0, 48.0)	49.0 (39.0, 58.0)	45.0 (39.0, 50.0)	53.0 (45.0, 59.0)	0.001
**Sex**					
Male	7 (5.5)	6 (4.7)	10 (7.9)	30 (23.6)	0.097
Female	21 (16.5)	11 (8.7)	15 (11.8)	27 (21.3)	
**Body mass index** (kg/m^2^)	21.4 (19.3, 22.5)	22.9 (21.4, 23.7)	27.3 (25.2, 30.5)	26.7 (23.7, 29.0)	<0.001
**Triglyceride levels** (mg/dL)	74.5 (59.3, 88.8)	61.0 (50.5, 99.0)	206.0 (148.0, 293.5)	181.0 (134.0, 293.0)	<0.001
**High density lipoprotein cholesterol levels** (mg/dL)	61.0 (58.3, 68.3)	65.0 (60.5, 73.0)	42.0 (36.5, 48.0)	45.0 (39.0, 55.5)	<0.001
**Low density lipoprotein cholesterol levels** (mg/dL)	107.0 (92.3, 123.0)	106.0 (90.5, 119.0)	149.0 (134.5, 164.5)	166.0 (143.0, 191.5)	<0.001
**Total cholesterol levels** (mg/dL)	182.0 (169.3, 193.8)	190.0 (157.5, 193.5)	219.0 (205.5, 243.0)	238.0 (218.5, 261.5)	<0.001
**Systolic blood pressure** (mmHg)	118.5 (109.5, 126.8)	115.0 (107.5, 122.5)	128.0 (117.0, 140.0)	136.0 (122.0, 141.5)	<0.001
**Diastolic blood pressure** (mmHg)	74.5 (69.5, 80.0)	70.0 (65.5, 76.5)	82.0 (75.5, 89.5)	86.0 (79.0, 92.0)	<0.001
**Fasting plasma glucose levels** (mg/dL)	89.0 (86.0, 93.5)	92.0 (89.5, 95.0)	100.0 (96.0, 118.5)	103.0 (97.0, 119.5)	<0.001
**Waist circumference** (cm)	75.5 (70.5, 79.0)	78.0 (73.5, 79.0)	91.0 (87.5, 95.0)	91.0 (86.0, 94.5)	<0.001
**Smoking habit**					
No	28 (22.0)	16 (12.6)	22 (17.3)	50 (39.4)	0.249
Yes	0 (0.0)	1 (0.8)	3 (2.4)	7 (5.5)	
**Alcohol consumption**					
No	22 (17.3)	11 (8.7)	17 (13.4)	39 (30.7)	0.501
Yes	4 (3.1)	3 (2.4)	7 (5.5)	17 (13.4)	
**Tooth brushing** (times/day)					
1	0 (0.0)	2 (1.6)	0 (0.0)	2 (1.6)	0.117
≥2	28 (22.0)	15 (11.8)	25 (20.0)	55 (43.3)	
**Education level**					
< Bachelor degree	9 (7.1)	10 (7.9)	7 (5.5)	28 (22.0)	0.068
≥ Bachelor degree	18 (14.2)	6 (4.7)	18 (14.2)	27 (21.3)	
**Exercise**					
Yes	12 (9.4)	6 (4.7)	19 (15.0)	41 (32.3)	0.020
No	12 (9.4)	8 (6.3)	3 (2.4)	11 (8.7)	
**Occupation**					
Government officer / Employee	11 (8.7)	6 (5.0)	12 (9.4)	22 (17.3)	
Commerce	8 (6.3)	3 (2.4)	7 (5.5)	18 (14.2)	0.780
Miscellaneous	9 (7.1)	8 (6.3)	6 (4.7)	17 (13.4)	
**Alveolar bone loss** (%)	14.5 (12.4, 17.5)	15.1 (11.9, 29.5)	15.8 (12.2, 17.7)	16.8 (13.3, 23.0)	0.027

* Qualitative data were analyzed using Chi–square.

Quantitative data with normal distribution (Low density lipoprotein cholesterol levels, systolic and diastolic blood pressure) were analyzed using ANOVA and the remaining data without normal distribution were analyzed by Kruskal–Wallis test.

### Plasma IgG to three periodontopathic bacteria and CRP levels

**Fig 1:**
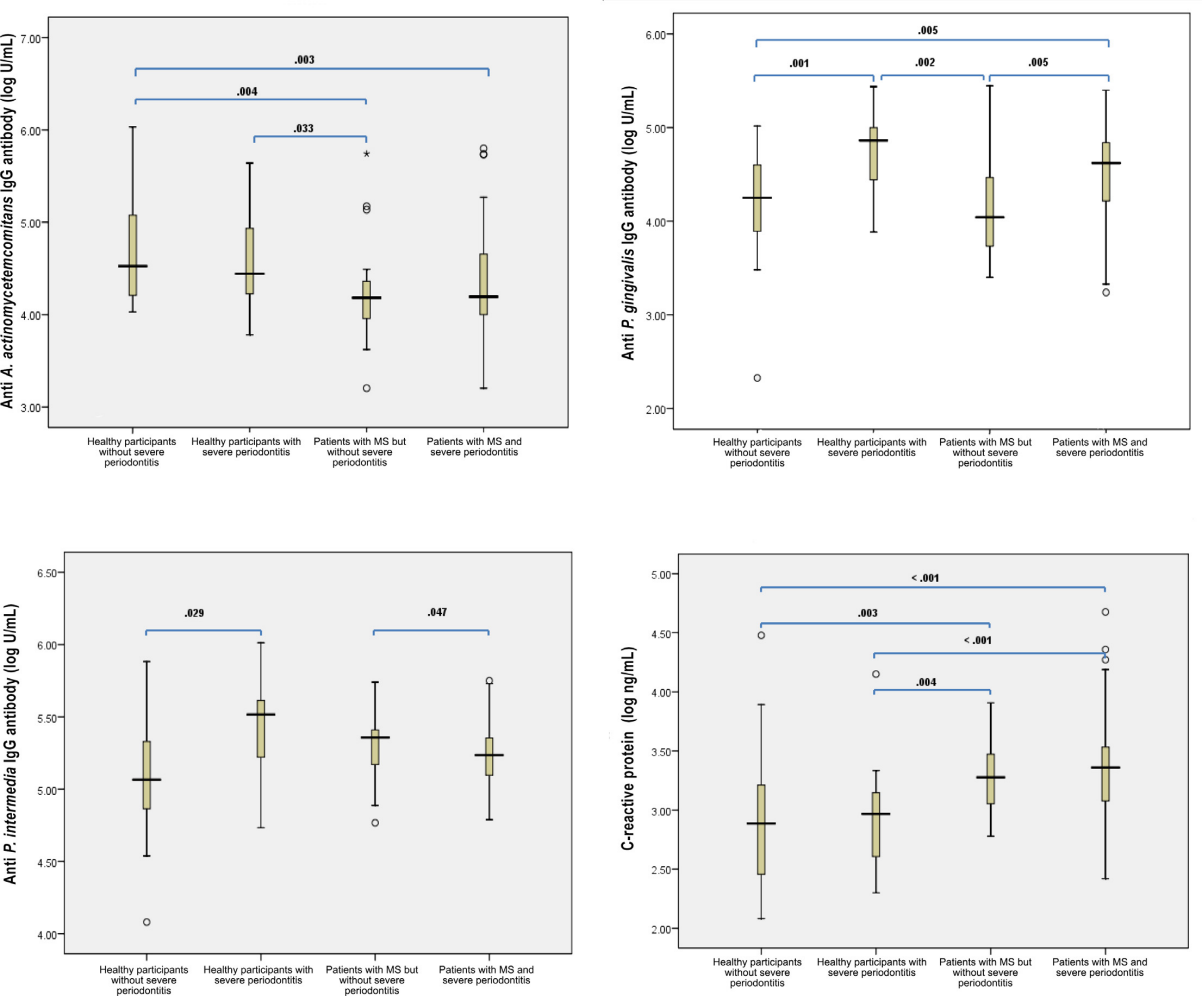
Levels of anti-periodontopathic bacteria IgG antibody and CRP according to the presence or absence of MS and severe periodontitis. Plasma IgG titers to *A*. *actinomycetemcomitans* were lower in patients with MS than in healthy participants, regardless of periodontal disease status (A). While plasma IgG titers to *P*. *gingivalis* were significantly higher in individuals with severe periodontitis than those without in both groups of healthy participants and MS patients (B), plasma IgG titers to *P*. *intermedia* did not show consistent findings with MS and periodontal status (C). CRP levels increased continuously from healthy participants without periodontitis to patients with MS and severe periodontitis (D).

Plasma IgG titers specific to the three periodontal pathogens and CRP levels are shown in Fig 1. Plasma IgG titers to *A*. *actinomycetemcomitans* were lower in patients with MS than those of healthy participants, irrespective of periodontal disease status [15,200.0 (8,379.4, 25,542.1) U/mL in patients with MS but absence of severe periodontitis and 15,614.2 (9,896.6, 43,673.6) U/mL in patients with MS and severe periodontitis; 33,562.2 (16,008.6, 96,113.8) U/mL in healthy participants without severe periodontitis and 27,779.2 (12,616.4, 118,020.0) U/mL in healthy participants with severe periodontitis]. While plasma IgG titers to *P*. *gingivalis* were significantly higher in individuals with severe periodontitis than those without in both groups of healthy participants and MS patients [73,004.4 (27,735.6, 99,861.2) U/mL in healthy participants with severe periodontitis compared to 17,874.6 (7,908.8, 40,503.6) U/mL) in healthy participants without severe periodontitis; 41,833.2 (15,854.4, 67,087.0) u/mL in patients with MS and severe periodontitis compared to 11,037.0 (5,255.4, 30,607.8) U/mL) in patients with MS but without severe periodontitis], plasma IgG titers to *P*. *intermedia* did not show consistent findings with MS and periodontal status. From all 127 subjects, a number of participants that IgG antibody for *A*. *actinomycetemcomitans*, *P*. *gingivalis* and *P*. *intermedia* could be detected were 120 (94.5%), 126 (99.2%) and 125 (98.4%) respectively. When compared using multiple regression, all periodontal parameters and a severe periodontitis status had a significant association with IgG antibody titers to *P*. *gingivalis* (*p*≤0.001) after adjusting for age, sex, smoking habit, alcohol consumption and education level (data not shown).

For plasma CRP levels, their positive titers were detected from all participants and the magnitude was highest in patients with MS and severe periodontitis (2,290.9 (1,189.6, 3,445.0) ng/mL), lower in patients with MS but without severe periodontitis (1,897.0 (1,138.8, 3,649.2) ng/mL), lower still in healthy participants with severe periodontitis (929.1 (404.0, 1,463.0) ng/mL), and lowest in healthy participants without severe periodontitis (770.5 (269.9, 1,658.4) ng/mL) (Fig 1). From a multiple linear regression analysis, a significant association was found between MS, mean PD, and CAL with CRP levels (β = 0.243, *p* = 0.036; β = 0.253, *p* = 0.006 and β = 0.025, *p* = 0.027, respectively). A marginally significant association was found for the mean percentage of BOP or periodontal disease status. None of the plasma levels of IgG to periodontopathic bacteria were associated with alveolar bone loss after adjusting for all periodontitis confounders. Alveolar bone loss was associated with age (β = 0.408, *p*<0.001), smoking habit (β = 0.243, *p* = 0.008) and CRP levels (β = 0.202, *p* = 0.024) (data not shown).

### Correlation of plasma IgG to three periodontopathic bacteria with CRP levels

**Fig 2:**
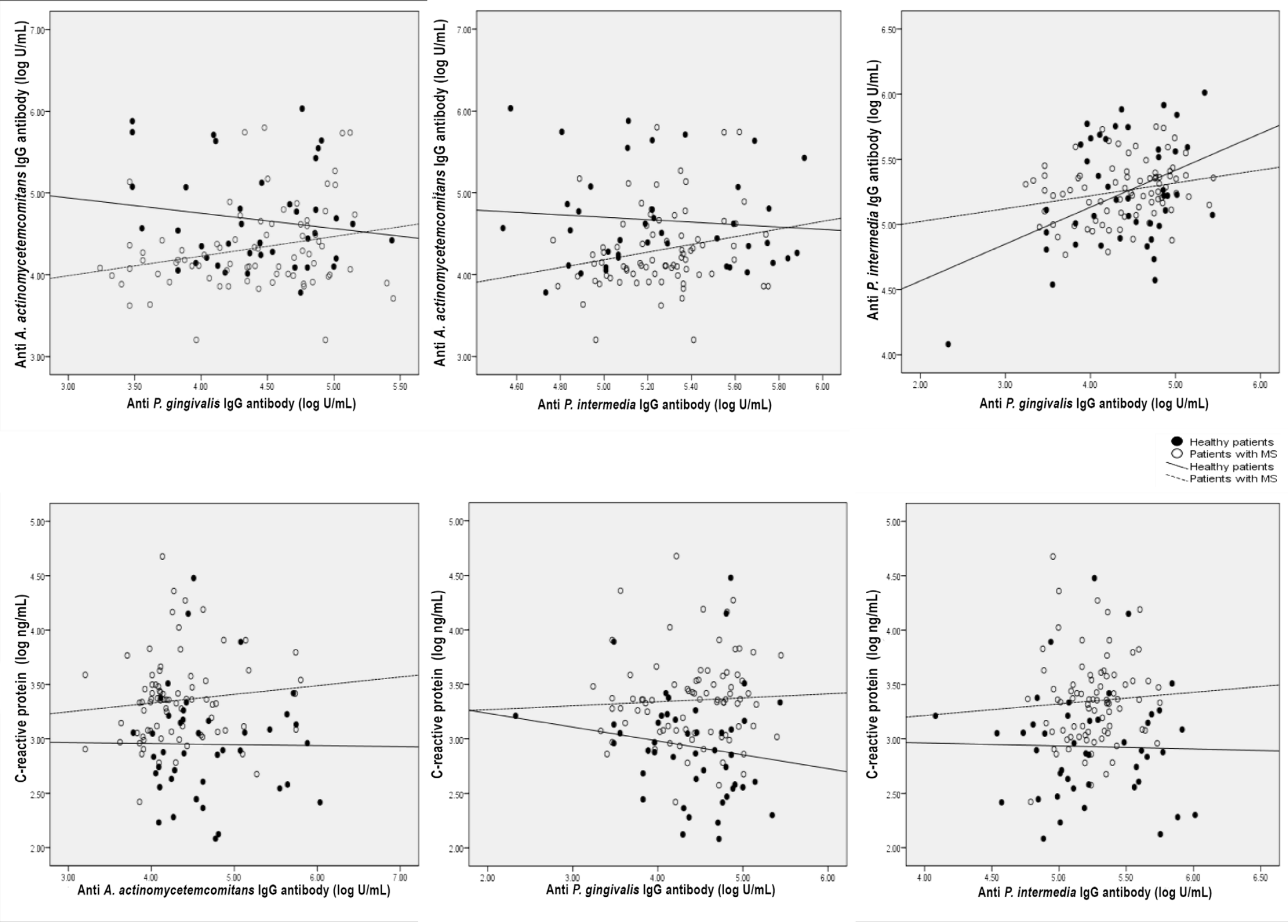
Correlation between anti-periodontopathic bacteria IgG antibody and CRP levels. Correlation coefficient (r) analyses between anti-*P*. *gingivalis* antibody (A); anti-*P*. *intermedia* antibody (B) and anti-*A*. *actinomycetemcomitans* antibody; anti-*P*. *intermedia* antibody and anti-*P*. *gingivalis* antibody (C); anti-*A*. *actinomycetemcomitans* antibody (D); anti-*P*. *gingivalis* antibody (E); anti-*P*. *intermedia* antibody (F) and CRP; controlled for the absence or presence of MS.

No statistically significant association was observed between CRP levels and IgG antibody titers to *A*. *actinomycetemcomitans*, *P*. *gingivalis* and *P*. *intermedia* (r = 0.050, *p* = 0.596; r = 0.010, *p* = 0.919; r = 0.056, *p* = 0.553, respectively). Among the IgG antibody titers for the three periodontal pathogens, while IgG antibody levels to *A*. *actinomycetemcomitans* and *P*. *gingivalis* or those of *A*. *actinomycetemcomitans* and *P*. *intermedia* were not correlated (r = 0.077, *p* = 0.414; r = 0.053, *p* = 0.572, respectively), IgG antibody levels to *P*. *gingivalis* were related to those of *P*. *intermedia* (r = 0.194, *p* = 0.037) after controlling for the absence or presence of MS (Fig 2).

### Amounts of salivary *A*. *actinomycetemcomitans* and *P*. *gingivalis*

**Fig 3:**
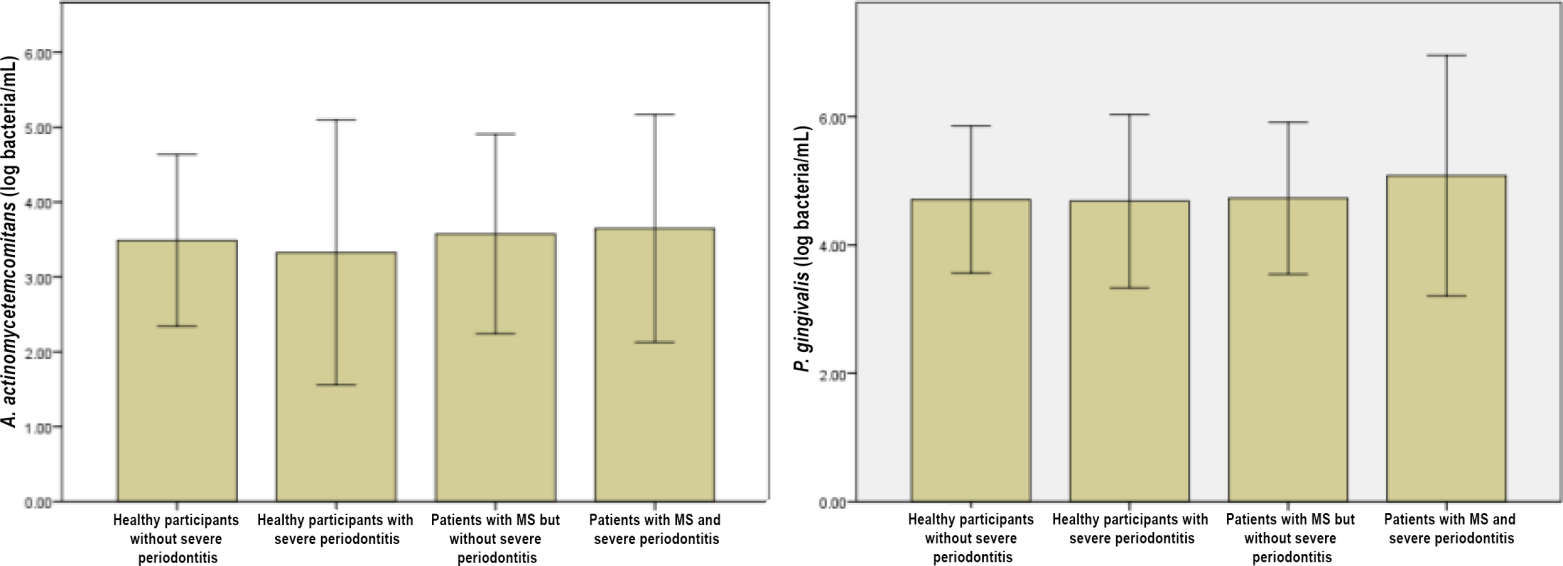
PCR analysis of salivary *A*. *actinomycetemcomitans* and *P*. *gingivalis*.

The results of the PCR analysis of saliva samples are shown in Fig 3. Although patients with MS and severe periodontitis tended to have higher salivary levels of *P*. *gingivalis* than those of other groups, *A*. *actinomycetemcomitans* and *P*. *gingivalis* did not differ quantitatively among healthy participants or patients with MS regardless of periodontal condition (*p* = 0.772 and *p* = 0.353, respectively).

### Correlation of salivary *A*. *actinomycetemcomitans* and *P*. *gingivalis* amounts and their IgG response levels

No significant correlation existed between salivary *A*. *actinomycetemcomitans* or *P*. *gingivalis* counts and their IgG titers in plasma both in healthy participants and patients with MS (r = 0.134, *p* = 0.424 and r = 0.229, *p* = 0.071, respectively).

### Association of plasma IgG to three periodontopathic bacteria and CRP levels with MS

Levels of CRP were significantly associated with the presence of MS and increasing components to four or five factors. Waist circumference was the only factor of MS that was associated with CRP levels. An inverse association was revealed between IgG antibody titers to *A*. *actinomycetemcomitans* and MS status whereas neither IgG antibody response to *P*. *gingivalis* nor *P*. *intermedia* was related to MS condition (Table 2).

**Table 2:** Association of MS and MS components with anti-periodontopathic bacteria IgG antibody and CRP levels as continuous measures adjusted for age, sex, BMI, smoking, alcohol consumption, education level, and exercise.

Model	Variables	Anti-*A*.*actinomycetemcomitans* IgG antibody		Anti-*P*. *intermedia* IgG antibody		Anti-*P*. *gingivalis* IgG antibody		CRP	
		β	*p*	β	*p*	β	*p*	β	*p*
**1**	**Metabolic syndrome:**								
	**presence**	−0.294	0.002		NS		NS	0.243	0.036
	r^2^	0.163		0.031		0.043		0.185	
	F for change in r^2^	9.634		4.174		5.500		4.498	
**2**	**Metabolic syndrome:**								
	**4–5 components**	−0.356	<0.001		NS		NS	0.260	0.027
	r^2^	0.185		0.031		0.043		0.190	
	F for change in r^2^	7.627		4.174		5.500		5.061	
**3**	**Components of MS:**								
	**WC**							0.391	0.007
	**FPG**					−0.221	0.032		
	**TG**	−0.315	0.001		NS				
	**HDL**					0.259	0.007		
	**SBP**	−0.262	0.005			0.369	<0.001		
	**DBP**								
	r^2^	0.258		0.031		0.143		0.208	
	F for change in r^2^	9.917		4.174		4.748		7.470	

NS: not significant

Plasma IgG titers to the three periodontal pathogens, CRP levels, severe periodontitis condition and confounders of MS were evaluated for the outcome of MS from final logistic regression models. Individuals with high CRP levels, high BMI, severe periodontitis status and low levels of plasma IgG to *A*. *actinomycetemcomitans* had a significantly higher association with MS than those who had high levels of plasma IgG to *A*. *actinomycetemcomitans*, low CRP levels, low BMI and healthy periodontal status (Table 3).

**Table 3:** Risk of MS by anti-periodontopathic bacteria IgG antibody and CRP levels.

Independent variables	Presence of MS							
			Model 1		Model 2		Model 3	
	Crude OR (95% CI)	*p*	Adjusted OR (95% CI)	*p*	Adjusted OR (95% CI)	*p*	Adjusted OR (95% CI)	*p*
**Age** (years)	1.1 (1.0–1.1)	0.034	1.1 (1.0–1.1)	0.062	1.0 (0.9–1.1)	0.466	1.0 (0.9–1.1)	0.692
**Sex: Female**	2.3 (1.1–5.1)	0.032	1.3 (0.4–4.0)	0.631	1.1 (0.3–5.0)	0.942	0.9 (0.2–4.0)	0.871
**Body mass index** (kg/m^2^)	2.4 (1.7–3.4)	<0.001	2.4 (1.7–3.3)	<0.001	2.6 (1.6–4.1)	<0.001	3.0 (1.7–5.2)	<0.001
**Plasma anti-*A*. *a* IgG Ab levels** (U/mL)								
**1st tertile**	1.0				1.0		1.0	
**2nd tertile**	0.4 (0.1–1.1)	0.080			0.1 (0.0–0.5)	0.009	0.1 (0.0–0.9)	0.040
**3rd tertile**	0.2 (0.1–0.6)	0.003			0.1 (0.0–0.6)	0.015	0.1 (0.0–0.7)	0.022
**Plasma anti-*P*. *I* IgG Ab levels** (U/mL)								
**1st tertile**	1.0				1.0		1.0	
**2nd tertile**	0.7 (0.3–1.8)	0.506			14.7 (1.3–66.5)	0.030	30.9 (1.7–548.8)	0.019
**3rd tertile**	3.6 (1.3–10.1)	0.013			1.8 (0.3–11.0)	0.542	2.8 (0.4–21.4)	0.312
**Plasma anti-P. g IgG Ab levels** (U/mL)								
**1st tertile**	1.0				1.0		1.0	
**2nd tertile**	1.2 (0.5–3.0)	0.649			0.4 (0.1–3.1)	0.410	0.3 (0.0–2.2)	0.210
**3rd tertile**	1.1 (0.5–2.7)	0.821			0.3 (0.1–2.5)	0.295	0.1 (0.0–1.3)	0.079
**Plasma CRP levels** (ng/mL)								
**1st tertile**	1.0				1.0		1.0	
**2nd tertile**	3.1 (1.3–7.4)	0.014			1.4 (0.3–7.4)	0.675	0.9 (0.2–5.4)	0.894
**3rd tertile**	8.8 (3.1–25.5)	<0.001			7.7 (1.0–59.4)	0.049	9.4 (1.0–88.2)	0.042
**Severe periodontitis: Presence**	3.8 (1.8–8.1)	0.001					7.8 (1.1–57.0)	0.044

## Discussion

This is the first study to identify plasma IgG response and salivary counts of periodontopathic bacteria as possible underlying associations between MS and periodontitis. We discovered that systemic exposure to major periodontal pathogens, *A*. *actinomycetemcomitans* (but not *P*. *gingivalis*), and signs of oral infection, severe periodontitis, as well as elevated CRP and BMI are associated with the presence of MS.

We report here that patients with MS had significantly lower IgG antibody responses to *A*. *actinomycetemcomitans* than healthy control participants, independent of salivary *A*. *actinomycetemcomitans* counts. In contrast, the antibody response to *P*. *gingivalis*, regardless of salivary levels, was significantly higher in severe periodontitis groups than in less-inflamed periodontal status groups. No apparent association was found between IgG antibody levels to *P*. *gingivalis* or *P*. *intermedia* and MS. No correlation was also observed between periodontopathic bacteria IgG antibody levels and CRP levels. There was a positive correlation between *P*. *gingivalis* and *P*. *intermedia* IgG antibody response, whereas there were no significant correlations found in the other paired comparisons.

The different patterns of IgG antibody response to *A*. *actinomycetemcomitans* and *P*. *gingivalis* in the current study suggest different relationships of the humoral immune response to periodontal pathogen infections in patients with MS. The antibody responses to periodontopathic bacteria are influenced by multiple factors beyond bacterial strains or periodontal status [11,18]. Although, we could not examine the prevalence of antibody-positive subjects because of no data of IgG antibody titer against periodontopathic bacteria in Thai patients with healthy periodontal status, causing no cut-off point for positive-negative border and further research is needed. Many host-related determinants and environmental factors have been proposed to differently modify or decrease antibody production. Age, race, sex, general health conditions, and smoking are related to variation in antibody production [10,19,20]. Therefore, in the current study, one of possible biological explanations for the association between MS and periodontitis may be related to modification of antibody production in MS. It could be hypothesized that the lack of elevation of IgG to *A*. *actinomycetemcomitans* in MS may result in less antibody protection in periodontitis. This in turn may increase periodontal disease severity irrespective of the normal response to *P*. *gingivalis* in chronic periodontitis. Graswinckel et al. observed decreased IgG levels in patients who smoke, irrespective of periodontal disease pathogens or severity [20]. However, in this study, the number of current smokers was small, such that the effect of smoking on antibody production could not be determined.

In one medical condition reported by Johansson et al.[21], a negative correlation between stroke in women and antibody response to *A*. *actinomycetemcomitans* was found, and MS was shown to correlate negatively with plasma IgG antibody levels to *A*. *actinomycetemcomitans* in this study. In general, higher plasma levels of IgG2, the predominant IgG in periodontitis and especially reactive with *A*. *actinomycetemcomitans*, are considered as protective to periodontal breakdown [22,23]. A lack of sufficient protective antibodies (in particular IgG2) against *A*. *actinomycetemcomitans* is perhaps in part responsible for increased severity of destructive periodontal disease in patients with MS. Offenbacher et al. proposed two host response axes that are important in periodontal disease: the T-cell-macrophage axis and the immunoglobulin-polymorphonuclear leukocyte axis [24]. *A*. *actinomycetemcomitans* seems to depend on a reduced humoral immune response, and in this situation, tissue damage may be mediated by the T-cell-macrophage axis. Another possible explanation is that elevated levels of CRP in MS may reduce T-cell responsiveness, resulting in a decrease of T-cell proliferation and T-cell-dependent antibody response. Furthermore, cells of the monocyte/macrophage lineage may be less activated, resulting in a decreased production of IL-1, IL-6, IFN-ϒ, and TNF-α, which are necessary for optimal production of IgG2 specific for *A*. *actinomycetemcomitans* [25–30]. Consequently, higher BMI and increased concentrations of CRP in patients with MS may render susceptibility to the *A*. *actinomycetemcomitans* response. The findings from this study appear to support the concept that the development of the plasma IgG antibody response to *A*. *actinomycetemcomitans* is protective. However, the pathway by which MS affects IgG production and consequently the pathogenesis of more severe periodontitis is not clearly understood.

Conversely, IgG antibody titers to *P*. *gingivalis* are positively correlated to periodontal disease severity. In this study, individuals with severe periodontitis, regardless of MS condition or count of *P*. *gingivalis* in saliva, have significantly higher titers of IgG to this pathogen compared with individuals without disease. These data confirm the strong association of *P*. *gingivalis* with periodontal disease severity in that immunoreactivity to *P*. *gingivalis* is frequent in adult chronic periodontitis [31]. *P*. *gingivalis* has been implicated as a periodontal pathogen and, similar to the present study, *P*. *gingivalis* elicits an elevated IgG antibody response that is strongly associated with chronic periodontitis [6,10,11,23,32]. Dye et al. investigated the ability of plasma IgG antibodies to periodontal bacteria to reflect clinical periodontal status. Comparable with the present study, they found that high plasma IgG titers to *P*. *gingivalis* combined with age, sex, smoking habit, education level and diabetes mellitus status were consistently associated with periodontitis, whereas titers to *A*. *actinomycetemcomitans* were not [6]. Titers of antibody levels can be used as an indicator of systemic bacterial exposure of periodontal disease, based on the rationale that persons with bacterial chronic exposure from periodontitis are likely to develop a systemic immune response, and IgG antibody levels are suitable markers for both present and past pathogen exposure, since they remain remarkably stable over a long period of time [33,34]. Lakio et al. reported that plasma IgG levels against *A*. *actinomycetemcomitans* and *P*. *gingivalis* were extremely stable during 15 years both in subjects with and without periodontitis [35]. Antibody levels were also reported to be associated with CHD, the final consequence of MS [36].

There are only two studies to date that investigated the levels of plasma IgG to *A*. *actinomycetemcomitans* or *P*. *gingivalis* in some groups of patients with MS [37,38]. Iwasaki et al. studied plasma IgG antibody to *P*. *gingivalis* in older Japanese adults with MS and revealed that participants with MS and increasing components were more likely to have an elevated plasma IgG antibody to *P*. *gingivalis* [37]. However, similar to the present study, Hyvärinen et al. did not find this association [38]. In contrast to the inverse association in the current study, significant seropositivity for *A*. *actinomycetemcomitans* in MS was found by Hyvärinen et al. [38]. This discrepancy may be due to the fact that these two studies were performed only in elderly or male patients. The use of certain periodontal parameters for clinical diagnosis of periodontitis (percentage of BOP or number of missing teeth), and the lack of bacterial counts to confirm this association may also have played a role. Therefore the current study provides a broader view of this research topic.

qRT-PCR is valuable for detection of periodontal pathogens and has a high sensitivity and specificity for bacterial quantification in dental plaque or salivary samples [39]. Strong correlations in *A*. *actinomycetemcomitans* and *P*. *gingivalis* counts across saliva and supra- and sub-gingival plaque have been found [40–42]. There are a few studies that have investigated periodontal pathogens in the Thai population [43–45].The prevalence of salivary *A*. *actinomycetemcomitans* detection in this study was 38.2%, and 57.3% for *P*. *gingivalis*. Counts of salivary *A*. *actinomycetemcomitans* and *P*. *gingivalis* among each group studied were not different. Some patients with healthy periodontal status can be presence of *P*. *gingivalis* because of the possible existence of different strains and genotypes. In the literature reported, a distribution may be noted, where the *FimA* II genotype is the most frequently found in patients with periodontitis, while in healthy patients the most frequent genotype is *FimA* I [46]. Moreover, the prevalence of each strain of *P*. *gingivalis* in the periodontal pockets of Thai population is not known. The PCR method used in this study is unable to identify sub-classification of the bacteria. Further studies are therefore needed to confirm these findings. Though prevalence of *A*. *actinomycetemcomitans* and *P*. *gingivalis* was dissimilar compared with other studies of the Thai population, this depends on bacterial detection methods, geographic location (rural or urban area), socioeconomic status, education level, and periodontal condition of study subjects. Red complex bacteria were predominant periodontal pathogens of the moderate to severe form of chronic periodontitis in a Thai population [43–45], and *A*. *actinomycetemcomitans* plays an important role in chronic periodontitis when coexisting within a red complex. Therefore, we studied the role of *A*. *actinomycetemcomitans* and *P*. *gingivalis* in our population. Salivary levels of each periodontopathic bacteria were not correlated with periodontal status in the current study, and previous reports supported these findings in that the presence of *A*. *actinomycetemcomitans* or *P*. *gingivalis* alone was not associated with periodontal disease status [40,43,44]. Paju et al. has shown that not only the species but also combinations of certain species or the number and amount of species in saliva are associated with periodontal parameters [47], which is in agreement with the present study.

Although a positive relationship between *A*. *actinomycetemcomitans* and *P*. *gingivalis* counts in saliva and the IgG antibody levels to these pathogens has been described previously [48], in this study, the circulating levels of IgG were unrelated to the amount of *A*. *actinomycetemcomitans* or *P*. *gingivalis* in saliva, similar to other previous studies [20,34,49]. A notion that may help to explain the present finding of no relationship between specific plasma antibody levels and the recovery of two test microorganisms is proposed. Antibodies may not necessarily decrease periodontal pathogen loads but may neutralize key virulence determinants, as well as promote opsonization, agglutination/precipitation, antibody-dependent cell-mediated cytotoxicity or complement activation [18]. Therefore, antibody response levels do not necessarily directly reflect the amount of exposure to periodontopathic infection. Accordingly, when we analyzed both aspects of the IgG antibody response and the amount of periodontal pathogens in this study, it could be postulated that the underlying association of MS with severe periodontitis might be from modulation of antibody response to periodontal pathogens, irrespective of their amount in the oral cavity. We thought that an altered IgG antibody response to *A*. *actinomycetemcomitans* and CRP may play a role in the association of MS and severe periodontitis in this group of the Thai population. Other serotypes of *A*. *actinomycetemcomitans* with unequal profiles of virulence as well as biological functions of IgG subclass and avidity investigation are required for diagnostic value. The validity assessment of these relationships in longitudinal study must also be further examined.

A number of studies have demonstrated that the CRP levels are higher in periodontitis patients than in control subjects [9–11,50]. The median level of CRP in periodontally healthy Thai individuals is 250 ng/mL compared to 650 ng/mL and 1,780 ng/mL in localized and generalized periodontitis respectively [50]. Moreover, many studies reported elevated CRP levels in patients with MS [7,8]. Our study reported positive titers in all participants and the CRP levels were elevated from lowest to highest in the following order: healthy participants without periodontitis, healthy individuals with periodontitis, patients with MS without severe periodontitis, and finally, patients with MS and severe periodontitis. Although an almost significant marginal association between CRP and periodontitis was observed in the present study, our findings suggest the systemic effect of local infection including periodontitis in the association between CRP and MS. Our data indicate that CRP levels were independent of *A*. *actinomycetemcomitans*, *P*. *gingivalis* and *P*. *intermedia* titers as inflammatory markers in patients with MS. The synergistic inflammatory response of periodontitis and MS may therefore contribute in part to the development of atherosclerosis, and ultimately lead to CHD.

The correlation of CRP levels with antibody titers to periodontopathic bacteria was positively demonstrated in some populations [51]. However, Miyashita [11] and the current study failed to demonstrate an obvious relationship between CRP and IgG levels. The relationship of CRP levels with IgG antibody titers to periodontopathic bacteria needs to be further clarified, because both markers show the systemic inflammatory response to periodontal infection and may potentially affect each other. The dissimilarities among these studies may be due to the different ethnicities, lifestyles, and health conditions of the participants, as well as the minimal size of the study population.

This study has some limitations. It is difficult to reveal a causal relationship among periodontal pathogens, systemic antibody response, periodontal disease, and MS, because this is a cross-sectional study. The number of subjects in this study might also be a limiting factor due to rigid inclusion criteria. Consequently, this study should be interpreted with some caution. Nevertheless, results of this study represent novel findings regarding the *A*. *actinomycetemcomitans* IgG antibody response to periodontitis were decreased in patients with MS. Future cohorts and a larger population with an integration of subject-level factors, microbial composition, and systemic or gingival tissue immune response, should be determined along with severity of periodontal disease expression to evaluate the underlying mechanism linking MS and periodontal disease.
